# Single-cell trajectory analysis of human homogenous neurons carrying a rare *RELN* variant

**DOI:** 10.1038/s41398-018-0177-8

**Published:** 2018-07-19

**Authors:** Yuko Arioka, Emiko Shishido, Hisako Kubo, Itaru Kushima, Akira Yoshimi, Hiroki Kimura, Kanako Ishizuka, Branko Aleksic, Takuji Maeda, Mitsuru Ishikawa, Naoko Kuzumaki, Hideyuki Okano, Daisuke Mori, Norio Ozaki

**Affiliations:** 1 Department of Psychiatry, Nagoya University Graduate School of Medicine, Nagoya, Aichi Japan; 2 Center for Advanced Medicine and Clinical Research, Nagoya University Hospital, Nagoya, Aichi Japan; 3 Institute for Advanced Research, Nagoya University, Nagoya, Aichi Japan; 4 Department of Physiology, Keio University School of Medicine, Shinjuku-ku, Tokyo Japan; 5 Department of Pharmacology, School of Pharmacy and Pharmaceutical Sciences, Hoshi University, Tokyo, Japan; 6 Brain and Mind Research Center, Nagoya University, Nagoya, Aichi Japan

## Abstract

Reelin is a protein encoded by the *RELN* gene that controls neuronal migration in the developing brain. Human genetic studies suggest that rare *RELN* variants confer susceptibility to mental disorders such as schizophrenia. However, it remains unknown what effects rare *RELN* variants have on human neuronal cells. To this end, the analysis of human neuronal dynamics at the single-cell level is necessary. In this study, we generated human-induced pluripotent stem cells carrying a rare *RELN* variant (RELN-del) using targeted genome editing; cells were further differentiated into highly homogeneous dopaminergic neurons. Our results indicated that RELN-del triggered an impaired reelin signal and decreased the expression levels of genes relevant for cell movement in human neurons. Single-cell trajectory analysis revealed that control neurons possessed directional migration even in vitro, while RELN-del neurons demonstrated a wandering type of migration. We further confirmed these phenotypes in neurons derived from a patient carrying the congenital RELN-del. To our knowledge, this is the first report of the biological significance of a rare *RELN* variant in human neurons based on individual neuron dynamics. Collectively, our approach should be useful for studying reelin function and evaluating mental disorder susceptibility, focusing on individual human neuronal migration.

## Introduction

Human genetic studies of patients with mental disorders such as schizophrenia (SCZ) have identified several possible factors contributing to neurodevelopmental impairments, one of which is a rare variant in the *RELN* gene encoding the glycoprotein reelin^1,2^. Reelin is well known as a controller of neuronal migration during brain development^3^. Indeed, carriers of homozygous *RELN* mutations exhibit lissencephaly accompanied by developmental delay^4^. Moreover, studies have suggested that even partial reduction in *RELN* mRNA and reelin protein may be related to several neurodevelopmental mental disorders^5–8^. In accordance with these genetic associations, *reeler* mice carrying *Reln* mutations demonstrate a defect in brain lamination and exhibit abnormal behaviors^3,9^. Nevertheless, the biological significance of rare *RELN* variants in human neurons remains unknown.

Neuronal migration is an essential event in the construction of a functional brain^10^. In vivo analysis using experimental animals has revealed strictly controlled mechanisms involved in neuronal migration^11,12^. Similar regulation seems to be present in developing human neurons; however, the single-cell dynamics of neuronal migration remains unexamined. Considering that sequential events occur in the developing brain, the analysis of live neurons is required for understanding neuronal dynamics relevant to neurodevelopmental events in humans.

We previously identified an inherited rare *RELN* variant (RELN-del) in one SCZ patient^1^. In this study, to better understand this RELN-del effect on human neurons, we generated isogenic RELN-del-induced pluripotent stem cell (iPSC) lines using targeted genome editing. Since tyrosine hydroxylase-positive (TH+) dopaminergic neurons express reelin during brain development in mice^13^ and have been considered one of the key factors in SCZ pathology^14,15^, we differentiated iPSCs into homogeneous dopaminergic neurons. Our single-cell analysis using live neurons revealed that healthy human neurons had controlled directional migration even at the single-cell level, while RELN-del neurons lost migration ability, particularly in directionality under the impaired reelin signal. We obtained a similar phenotype using neurons derived from subjects carrying congenital RELN-del. Finally, our automatic evaluation system of the migration of individual neurons confirmed that RELN-del triggers sequential disruption of directional migration.

## Materials and methods

### Subjects

The human female iPSC line 201B7 (HPS0063)^16^ was provided by RIKEN BRC as one of the controls (CON1). Furthermore, two healthy Japanese subjects, a 51-year-old male (CON2) and a 41-year-old male (CON3), were selected as sources for control iPSCs. We previously identified two subjects with inherited heterozygous RELN-del (chr7: 102919640–102930809, NCBI37/hg18)^1^: a 58-year-old Japanese male diagnosed with SCZ (RELN1) and his 83-year-old mother (RELN2) without SCZ. All subjects provided written informed consent. The given ages of the subjects are those at the time of the blood sampling for iPSC generation.

### iPSC selection

Chromosomal aneuploidy in iPSCs may be an unavoidable side effect of the reprogramming process^17^. To preclude the influence of aneuploidy on subsequent results, we excluded iPSC lines with unexpected copy number variations (CNVs). Although the iPSC line derived from CON2 harbored a 20q11.21 duplication, it was used for further analysis because this CNV has been frequently detected in human embryonic stem cells and iPSCs^18,19^. As a result, the used numbers of our generated iPSC lines in this study were as follows: CON2 subclone 1, CON3 subclone 1, RELN1 subclone 1–3, and RELN2 subclone 1. The results of these lines are shown in Supplementary Table 1. We confirmed no clinically significant CNVs^1^ in the genomic DNA from CON1.

### Neuronal differentiation

Neuronal differentiation was induced as previously reported^20^, with some modifications. To obtain neurospheres, iPSCs pretreated with SB431542 (3 μM), CHIR99021 (3 μM), and dorsomorphin (3 μM) for 1 week (days 0–7) were dissociated using TrypLE select and cultured in neurosphere medium consisting of MHM (DMEM/F12 supplemented with 1× N2, 0.6% glucose, 100 units/ml penicillin, 100 μg/ml streptomycin, 5 mM HEPES) plus 1× B27, 20 ng/ml bFGF, 10 ng/ml hLIF, 10 μM Y-27632, 3 μM CHIR99021, 2 μM SB431542, 100 ng/ml FGF8, and 1 μM purmorphamine for 2 weeks (days 7–21). At day 14, neurospheres were dissociated for passage. For induction of dopaminergic neurons, secondary neurospheres were collected 1 week after passage (day 21) and plated onto Matrigel (BD)- or poly-l-ornithine/laminin-coated dishes in dopaminergic neuron medium (MHM supplemented with B27, 10 μM DAPT, 20 ng/ml BDNF, 20 ng/ml GDNF, 0.2 mM ascorbic acid, 1 ng/ml TGF-β3, and 0.5 mM dbcAMP). Cells were cultured in a 5% CO_2_/18–22% O_2_ atmosphere during all experiments.

### Evaluation of neuronal migration

Migration assays were performed according to a previous study^21^ with some modification. Briefly, secondary neurospheres were manually collected after 1 week (day 21, defined as the beginning of neuronal induction) and cultured on Matrigel-coated plates in dopaminergic neuron medium. Time-lapse movies were acquired using IncuCyte (Essen Bioscience, USA). For manual cell tracking, each sequential image at 15-min intervals from 48 to 52 h after seeding in dopaminergic neuron medium was examined by the Cell-Tracking application in ImageJ, as described previously^22^. The migration distance was measured by the *XY* coordinates from the origin. Plots of cell trajectories emanating from the origin, and the directionality ratio over time, were analyzed as previously reported^23^.

For the analysis of migration angles, the position of each cell in the *XY* coordinate was converted to the movement angle from the reference position as an inverse tangent (tan^−1^) of *X* and *Y* using MATLAB (Mathworks, Natick). The cell position in image *n* was termed Cell^*n*^. Cell^0^ represented the cell position at the starting time (defined as 48 h after the beginning of induction) and set as the origin. The reference direction of the cell was obtained by averaging the movement angles over the 4 h. The movement angle of a cell at each time point thereafter was expressed as the angle from the coordinate axis. Migration in a direction close to the reference direction (small angle) implies orientated migration while veering from the reference direction (larger angle) implies disorientated migration. When a cell did not move between two sequential images, the data at that time point were omitted from the analysis.

For the automatic detection of neuronal migration, secondary neurospheres were labeled using CellLight Nucleus-GFP (Thermo Fisher, USA). Automatic cell tracking was carried out by the Particle Tracker application in ImageJ^24^ using each sequential image at 15-min intervals from 42 to 54 h after plating in dopaminergic neuron medium. GFP signals, which could be detected at all-time points, were used for further examination. To reduce detection errors, GFP signals in aggregates were omitted from the analysis.

### Statistical analysis

Two means were compared using the Student’s or Welch’s *t-*test (unpaired, two tailed). Three or more means were analyzed by analysis of variance, followed by Dunnett’s test for pairwise comparisons. A *p* < 0.05 was accepted as a significant difference. Frequencies and ratios were compared by Pearson’s chi-squared test with *p* < 0.05 accepted as significant.

## Results

### Establishment of isogenic RELN-del iPSCs by targeted genome editing

**Fig. 1:**
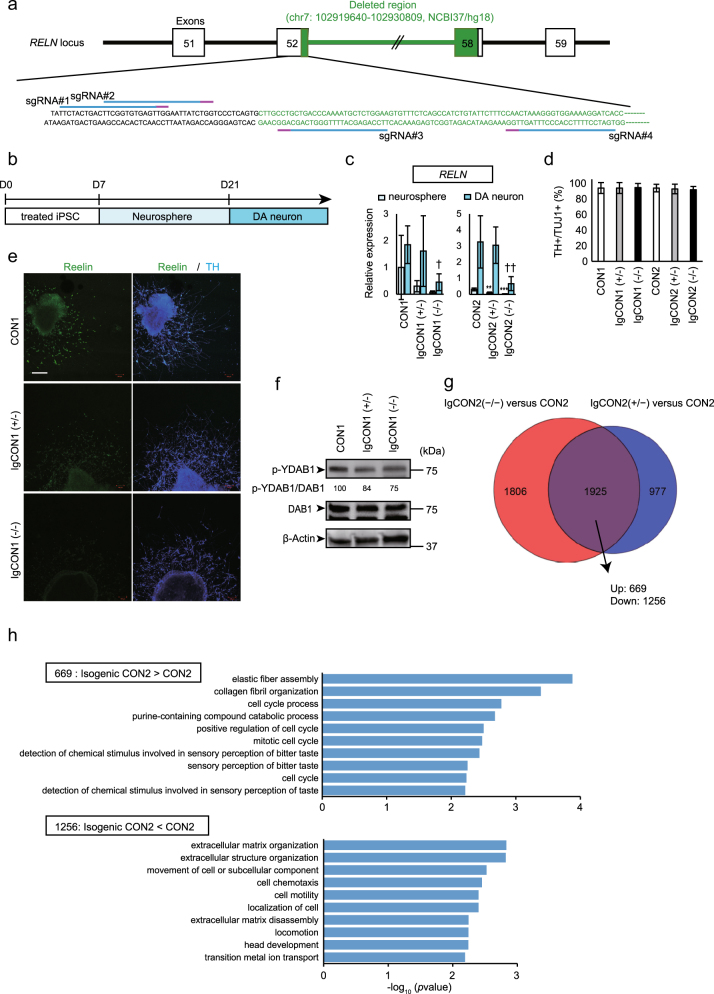
Characterization of isogenic RELN-del iPSCs and neurons. **a** The target sites of the CRISPR-sgRNAs used in this study. CRISPR-sgRNA#1 and #2 target the sense strand, whereas sgRNA#3 and #4 target the antisense strand. **b** Schematic illustration of the differentiation of dopaminergic (DA) neurons. **c** Quantitative real-time PCR was performed to measure *RELN* expression in neurospheres (day 21) and dopaminergic neurons (day 28). CON1: neurosphere *n* = 4, DA neuron *n* = 5. CON2: neurosphere and DA neuron *n* = 4. IgCON1(+/−): neurosphere *n* = 4, DA neuron *n* = 3. IgCON1(−/−): neurosphere *n* = 3, DA neuron *n* = 4. IgCON2(+/−): neurosphere *n* = 3, DA neuron *n* = 6. IgCON2(−/−): neurosphere *n* = 4, DA neuron *n* = 5. Bars represent the mean ± SD. **p < 0.01, ***p < 0.001 vs respective parental neurospheres. ^†^*p* < 0.05, ^††^*p* < 0.01 vs respective parental DA neurons. **d** Analysis of dopaminergic neuron differentiation efficiency by quantifying the ratio of TH+ to TUJ1+ cells at day 23 (*n* = 180 cells). Bars represent mean ± SD. **e** Representative images of early dopaminergic neurons (day 23 = 48 h after the start of induction) immunostained for TH and reelin. The white bar in the image represents 200 μm. **f** Immunoblotting for phosphorylated tyrosine DAB1 (p-YDAB1), total DAB1, and β-actin using DA neurons at day 28. The values of p-YDAB1/total DAB1 are as follows: CON1 = 100, IgCON1(+/−) = 84, and IgCON1(−/−) = 75. **g** The venn diagram containing the altered gene expression in IgCON2(+/−) (blue) or IgCON2(−/−) (red). **h** GO analysis identified enriched categories for the altered genes in both IgCON2 neurospheres. A list of the top 10 categories showing the smallest *p*-values

We previously identified an inherited rare *RELN* variant, the heterozygous *RELN* deletion (RELN-del) (chr7: 102919640–102930809, NCBI37/hg18), in a patient with SCZ^1^. To understand the biological effect of this RELN-del on human neuronal cells, we generated isogenic RELN-del iPSC lines using the CRISPR/Cas9 system. Four individual single-guide RNAs (sgRNAs) were constructed near the start point of the deleted region in the patient with SCZ (Fig. 1a). Among the constructed sgRNAs, sgRNA#4 showed the strongest cleavage activity as examined by T7EI assay (Supplementary Fig. 1a). To achieve the reproducibility of results and to exclude the possibility of off-target effects, we generated multiple isogenic iPSC lines from the two parent iPSC lines (CON1 and CON2) as previously suggested^25^. We obtained several isogenic lines with heterozygous or homozygous RELN-del (Supplementary Fig. 1b). Each isogenic line was designated as Ig-(parent iPSC name)-genotype); for example, IgCON1 (+/−) is the heterozygous isogenic line derived from CON1 iPSCs. These isogenic iPSC lines retained the capacity to differentiate into all three germ layers (Supplementary Fig. 1c). We also performed an off-target search using CCTop^26^. CCTop predicted 10 potential off-target sites for sgRNA#4 in the human genome under the following conditions: core length = 12; max. core mismatches = 2; max. total mismatches = 3. However, the only target at an exonic position was in the *RELN* gene region (Supplementary Table 2).

### Characterization of isogenic RELN-del neuronal cells

To test whether the generated isogenic RELN-del lines demonstrate decreased reelin expression levels, we first examined *RELN* messenger RNA (mRNA) expressions on day 21 (containing predominantly neuronal progenitors or stem cells) and in differentiated dopaminergic neurons on day 28. A differentiation scheme is shown in Fig. 1b. Compared with parental lines, the isogenic homozygous RELN-del lines showed much less expressions of *RELN* mRNA (Fig. 1c). There were no differences in the rate of differentiation into TH+ cells among all the groups (>85%; Fig. 1d).

We next performed immunocytochemistry for reelin in TH+ dopaminergic neurons. To facilitate the direct comparison of reelin immunoexpression among neurons derived from different iPSC lines (CON1, IgCON1[+/−], IgCON1[−/−]), staining was conducted simultaneously. The highest reelin immunoexpression was detected in TH+ dopaminergic neurons derived from parental iPSC CON1. Weaker expression was detected in neurons derived from CON1(+/−), and no detectable expression was observed in IgCON1(−/−)-derived neurons (Fig. 1e). These findings indicate that reelin expression in isogenic RELN-del dopaminergic neurons is directly dependent on genetic insufficiency, and the decreased reelin expression may cause the impaired reelin signal activity. The phosphorylation of disabled-1 (DAB1)^27^ tyrosine is one of the reelin signals required for neurodevelopment. As shown in Fig. 1f, the amount of phosphorylated DAB1 tyrosine (p-YDAB1) was decreased in the isogenic RELN-del cell lines compared with that in parental lines, indicating that the reelin signal is impaired in isogenic RELN-del neurons.

To characterize the isogenic RELN-del neuronal cells, we compared the gene expression profiles of isogenic RELN-del and their parental neurospheres (Fig. 1g, Supplementary Table 3), considered to be a direct effect of RELN-del. The upregulated genes in isogenic RELN-del neurospheres were mainly associated with the cell cycle (Fig. 1h upper, Supplementary Table 4). In contrast, the downregulated genes in isogenic RELN-del neurospheres were enriched in cell movement GO terms (Fig. 1h lower, Supplementary Table 5).

### SCZ patient-derived RELN-del impairs the directionality of dopaminergic neuronal migration

**Fig. 2:**
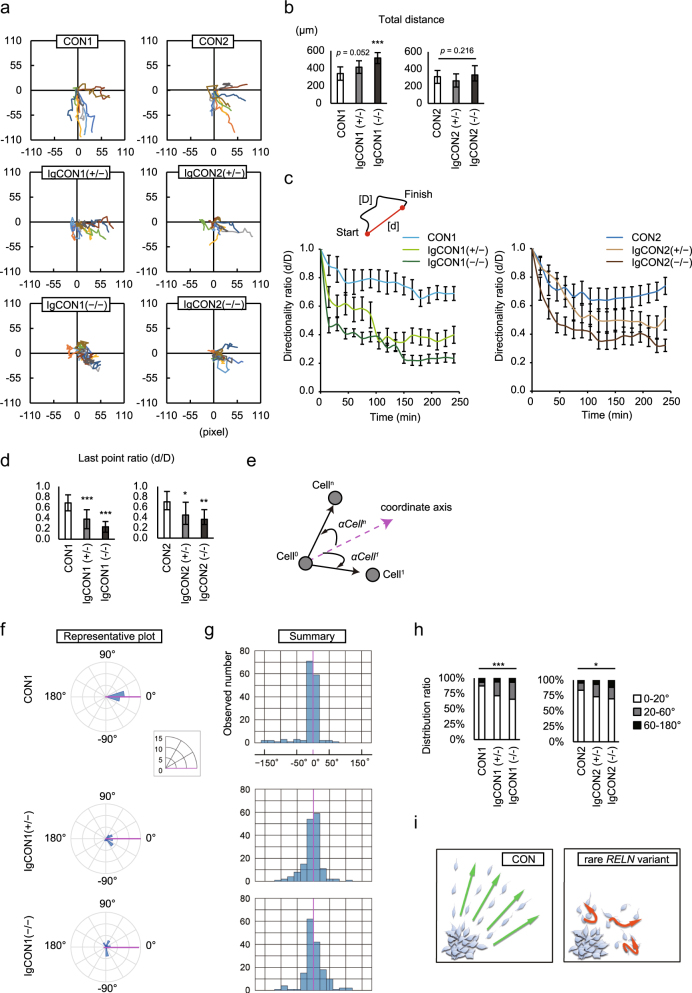
Loss of directed migration trajectory in isogenic RELN-del neurons. **a** Plots of 10-cell trajectories emanating from the origin. 1 pixel = 3.42 μm. **b** Quantification of total migration distance over 4 h (48–52 h after plating). Bars represent the mean ± SD. ***p < 0.001. *n* = 10. **c** *Upper*: Definition of directionality ratio. *Lower*: Directionality ratio over elapsed time. Plots represent the mean ± SEM. *n* = 10. **d** Directionality ratio of the last point trajectory. Bars represent mean ± SD. *p < 0.05, **p < 0.01, ***p < 0.001. *n* = 10. **e** Schematic diagram showing the cell movement angle at each time point *n* relative to the coordinate axis. *αCell*^*n*^ = angle for Cell^*n*^. **f** Distribution of the cell movement angles for each genotype plotted on circular histograms. Each histogram shows a representative cell for each genotype. The diameter represents the cell numbers. **g** Summary of 10 cells from each genotype shown as a histogram. **h** The distribution of cells based on their movement angles. Angles are absolute values relative to the origin (negative is clockwise). For example, 0°−20° include both *αCell*^*n*^ = 0°−20° and −20°–0°. Each bar represents the proportion of the total analysis points according to the angular movement range 0°–20°, 20°–60°, and 60°–180° for each genotype. CON1 *n* = 149, CON2 *n* = 163, IgCON1(+/−) *n* = 159, IgCON1(−/−) *n* = 159, IgCON2(+/−) *n* = 145, IgCON2(−/−) *n* = 152. *p < 0.05, ***p < 0.001. **i** Graphical abstract

To examine whether isogenic RELN-del neurons exhibited impaired cell movement as a result of GO analysis, we performed a single-cell-tracking assay, which makes it possible to capture individual cell movement. Neurons derived from parental iPSCs (CON1 and CON2) migrated straight toward the outside, while those from isogenic RELN-del lines exhibited circuitous trajectories (Fig. 2a, Supplementary Fig. 2). For an objective indicator of directionality, we compared quantitative indices of migration between parental lines and isogenic RELN-del lines. Comparing total migration distance over 4 h from 48 to 52 h after dopaminergic induction, IgCON1 (+/−), IgCON2 (+/−), and IgCON2 (−/−) did not show significant differences from each parental line, although IgCON1 (−/−) demonstrated a larger migration distance (Fig. 2b). The parameter of directionality ratio, the straightline length between the start point and the endpoint of the migration trajectory divided by the length of the trajectory, showed sequentially lower values in isogenic RELN-del line neurons than their parental line neurons during the observed time. This corresponded with decreasing reelin expression (Fig. 2c, d).

The directional ratio is sometimes influenced by cell speed^23^. To preclude this, we also measured the stability of each cell movement angle. Cells with greater directional movement will show smaller angles (i.e., straighter trajectories) compared with cells with disorientated (circuitous) movement. Measurement metrics are summarized and schematically depicted in Fig. 2e. The movement angle for Cell^*n*^ (*αCell*^*n*^) is defined as the angle of the displacement vector for Cell^*n*^ from the coordinate axis, with positive angles indicating counter-clockwise motion and negative angles indicating clockwise motion. As shown in Fig. 2f, g and Supplementary Fig. 3, the movement angles in parental lines were usually less than 20° (−20° < *αCell*^*n*^ < 20°; 87% of the total for CON1, 83% for CON2). However, fewer angles were within this range for isogenic RELN-del groups (71% of the total for IgCON1 [+/−], 65% for IgCON1[−/−], 73% for IgCON2[+/−], and 70% for IgCON2[−/−]). The distribution ratio based on the movement angles in isogenic RELN-del groups was significantly different from parental groups (Fig. 2h). Taken together, it appeared that RELN-del causes the disruption of directed migration in human neurons at the single-cell level, and this effect was dependent on reelin dosage (Fig. 2i).

### Characterization of congenital RELN-del iPSCs and neurons

**Fig. 3:**
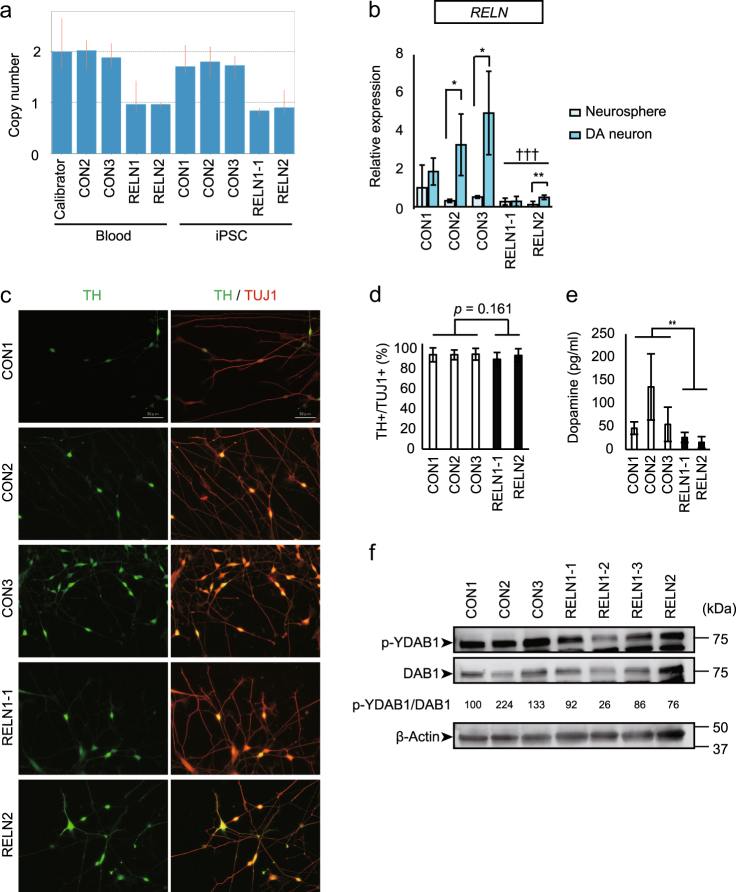
Generation of iPS cells carrying congenital RELN-del. **a** Confirmation of genomic *RELN* deletion in RELN-del iPSCs by the TaqMan copy number assay. Calibrator = a sample carrying two copies of *RELN*. **b** Quantitative real-time PCR was performed to measure *RELN* expression in neurospheres (day 21) and dopaminergic neurons (day 28). CON1: neurosphere *n* = 4, DA neuron *n* = 5. CON2: neurosphere and DA neuron *n* = 4. CON3: neurosphere and DA neuron *n* = 5. RELN1-1: neurosphere *n* = 4, DA neuron *n* = 6. RELN2: neurosphere *n* = 4, DA neuron *n* = 6. Bars are mean ± SD. *p < 0.05, **p < 0.01, ^†††^*p* < 0.001 (DA neuron: CON vs RELN-del). The results of CON1 and CON2 were cited from Fig. 1c. **c** Representative images of early dopaminergic neurons (day 23 = 48 h after the start of dopaminergic neuron induction), followed by immunostaining for TH and TUJ1. Bars represent 50 μm. **d** Analysis of dopaminergic neuron differentiation efficiency by quantifying the ratio of TH+ to TUJ1+ cells at day 23 (*n* = 180 cells). Bars represent the mean ± SD. The results of CON1 and CON2 were cited from Fig. 1d. **e** Measurement of dopamine concentration in the culture supernatant of dopaminergic neurons at day 28. CON1 *n* = 5, CON2 *n* = 4, CON3 *n* = 3, RELN1-1 *n* = 5, RELN2 *n* = 4. Bars represent mean ± SD. **p < 0.01. **f** Immunoblotting for phosphorylated DAB1 tyrosine (p-YDAB1), total DAB1, and β-actin using DA neuron at day 28. The relative intensities of p-YDAB1/total DAB1 are as follows: CON1 = 100, CON2 = 224, CON3 = 133, RELN1-1 = 92, RELN1-2 = 26, RELN1-3 = 86, and RELN2 = 76

To investigate whether undirected migration is also observed in congenital RELN-del neurons, we generated iPSC lines from the peripheral blood monocytes of two congenital heterozygous RELN-del subjects, which we previously identified^1^ (RELN1, the patient with SCZ, and RELN2, his healthy mother). To confirm the heterozygous deletion of *RELN* in congenital RELN-del iPSC lines, we performed TaqMan copy number assays. As shown in Fig. 3a, RELN-del was detected in iPSC lines as well as in blood monocytes from the source subjects (RELN1 and RELN2).

We examined *RELN* mRNA expression in neurospheres on day 21 and in differentiated dopaminergic neurons on day 28, using congenital RELN-del lines. Dopaminergic neurons differentiated from control iPSCs showed elevated *RELN* mRNA expression compared with progenitor/stem cells in neurospheres derived from the same iPSCs (Fig. 3b), indicating that dopaminergic neuron differentiation is associated with increased reelin expression. In contrast, dopaminergic neuronal cells derived from congenital RELN-del iPSCs exhibited significantly reduced *RELN* mRNA expression compared with controls (Fig. 3b).

Previous studies have reported that iPSCs derived from SCZ patients differ in their capacity to differentiate into dopaminergic neurons and produce dopamine compared with iPSCs derived from healthy controls^28,29^; however, this remains controversial. Under our induction protocol, all iPSCs, including RELN-del iPSCs from the patient with SCZ (RELN1), demonstrated a high capacity to differentiate into TH+ cells (over 85%) (Fig. 3c, d). This made it possible to analyze relatively homogenous neurons. In contrast, dopaminergic neurons derived from the congenital RELN-del iPSCs (RELN1-1 and RELN2) released substantially less dopamine compared with neurons derived from control iPSCs (Fig. 3e).

To address whether each congenital RELN-del TH+ neuron showed an impaired reelin signal similar to isogenic RELN-del line neurons, we performed immunoblotting experiments for p-YDAB1 and total DAB1. The amount of p-YDAB1 was decreased in congenital RELN-del lines compared with control lines (Fig. 3f). These findings confirm that this rare *RELN* variant causes decreased reelin expression and impaired reelin signaling in human neurons.

**Fig. 4:**
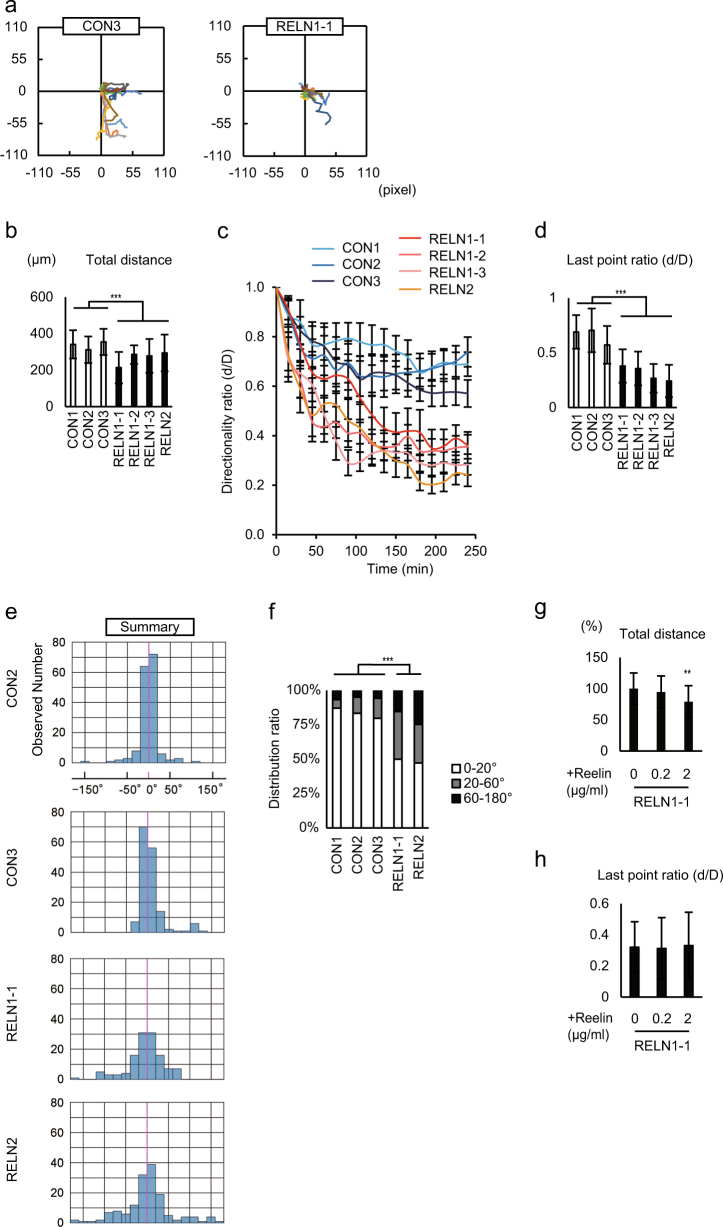
Congenital RELN-del dopaminergic neuron shows migration defects. **a** Plots of 10-cell trajectories emanating from the origin. 1 pixel = 3.42 μm. **b** Quantification of total migration distance over 4 h (48–52 h after plating). Bars represent the mean ± SD. ***p < 0.001. *n* = 10. **c** Directionality ratio over elapsed time. Plots represent the mean ± SEM. **d** Directionality ratio of the last point trajectory. Bars represent the mean ± SD. ***p < 0.001. *n* = 10. **e** Summary of 10 cells from each genotype shown as a histogram. **f** The distribution of cells based on their movement angles. CON1 *n* = 149, CON2 *n* = 163, CON3 *n* = 158, RELN1-1 *n* = 124, RELN2 *n* = 150. ***p < 0.001. **g** Effect of recombinant reelin on migration distance. **p < 0.01 vs RELN1-1 without recombinant reelin. **h** Effect of recombinant reelin on migration directionality. The results of CON1 and CON2 were cited from Fig. 2

Next, we performed a live cell-tracking analysis using congenital RELN-del lines. Dopaminergic neurons derived from healthy control iPSCs (CON1, CON2, and CON3) migrated straight toward the outside, while those from congenital RELN-del iPSCs (RELN1 and RELN2) exhibited circuitous trajectories (Fig. 4a and Supplementary Figs. 2 and 4). Unlike isogenic RELN-del neurons, congenital RELN-del neurons lost the mobilization capacity for both distance and direction (Fig. 4b–d). Single-cell analysis of movement angles indicated that congenital RELN-del neurons severely lost the directionality of migration (Fig. 4e, f and Supplementary Figs. 3 and 5).

To investigate whether the impaired migration in distance and directionality was due to the reduction of reelin secretion in congenital RELN-del lines, we performed experiments using recombinant reelin protein. Although the exogenous reelin induced more impairment of the neuronal migration distance, it had no effect on neuronal directionality at the single-cell level (Fig. 4g, h).

### Automatic evaluation system using single-cell trajectory analysis

**Fig. 5:**
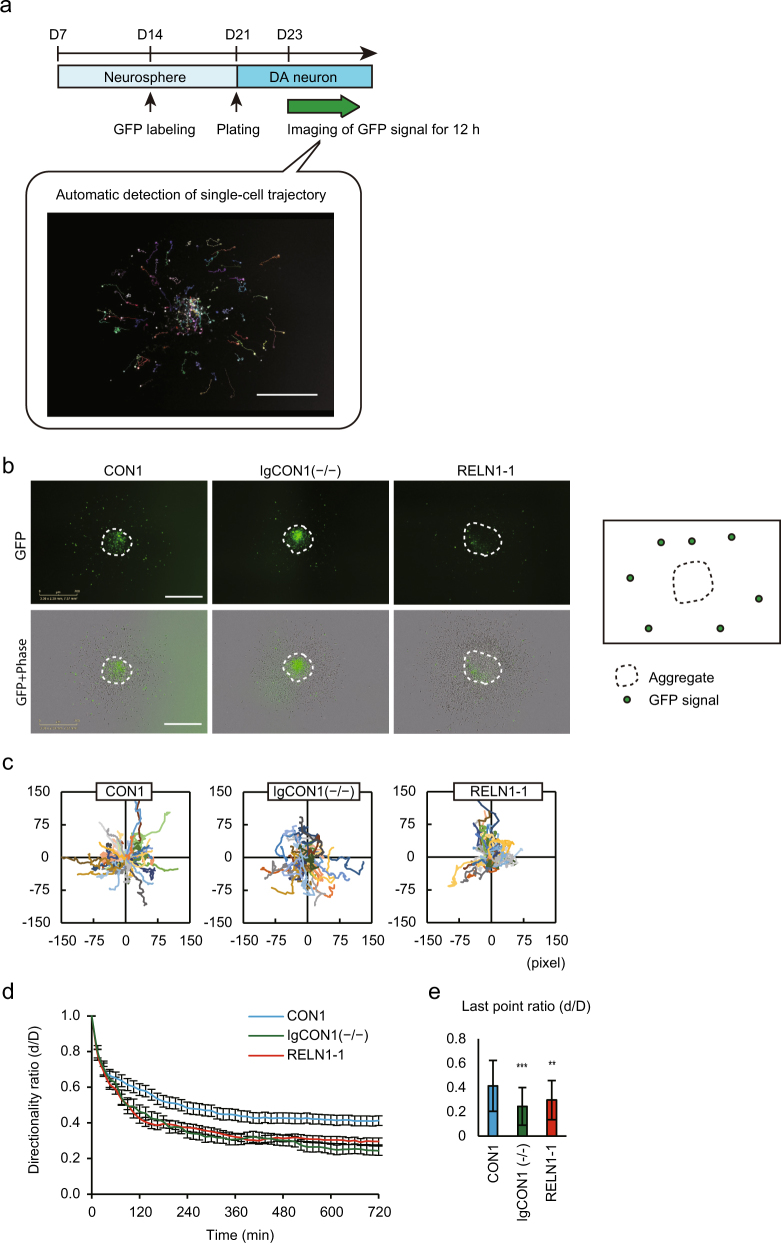
Automatic detection system for neuronal directionality. **a** *Upper:* Schematic illustration of the automatic detection system. *Lower:* A representative result of automatic detection. **b** *Left*: Images of GFP signal at 48 h after plating neurospheres. The white bars in images are 700 μm. *Right*: Schematic diagram of left images. **c** Plots of cell trajectories emanating from the origin. 1 pixel = 3.06 μm. CON1 *n* = 58, IgCON1(−/−) *n* = 37, RELN1-1 *n* = 70. **d** Directionality ratio over elapsed time. Plots represent the mean ± SEM. CON1 *n* = 58, IgCON1(−/−) *n* = 37, RELN1-1 *n* = 70. **e** Directionality ratio of the last point trajectory in **d**. Bars represent the mean ± SD. **p < 0.01, ***p < 0.001 vs CON1

We speculate that our analysis method is worthy for screening neuron dynamics at the single-cell level without any bias. We then performed single-cell trajectory analysis with an automatic detector using a GFP signal for 12 h (Fig. 5a). The trajectory of each neuron could be tracked by GFP signals (Fig. 5b). Compared with control neurons (CON1), RELN-del neurons (IgCON1[−/−] and RELN1-1) showed meandering migration trajectories (Fig. 5c). Consistent with the manual tracking results, automatic analysis showed that the migration directionality decayed with decreasing reelin expression (Fig. 5d, e).

## Discussion

The inaccessibility of the living human brain is a major obstacle to elucidate neuronal function in humans and the pathogenesis of mental disorders. Recently, iPSCs have made it possible to examine living human central nervous system neurons. Human iPSC-derived neurons show gene expression patterns similar to those in the developing human brain^30^ and reflect their various traits in vitro^31–33^. Considering that reelin functions well in the developing brain, the establishment of RELN-del iPSCs is an ideal strategy to assess the significance of rare *RELN* variants in human neurons.

The reelin-dependent signaling pathway is involved in brain function, particularly for brain development. One of the major reelin signals is the phosphorylation of DAB1, which is required for neuronal migration^34^. We showed that both isogenic and congenital RELN-del neurons demonstrate a reduction in p-YDAB1. Therefore, this *RELN* variant causes an impaired reelin signal in neurons, resulting in a disruption in directional migration. A number of previous studies have strongly suggested that the genetic anomaly of *RELN*^35–37^ and impaired reelin signals^7,38,39^ are vulnerable factors for SCZ. Thus, it is reasonable to assume that the disrupted directional migration triggered by RELN-del may be relevant for SCZ. Moreover, we found that the addition of recombinant reelin was ineffective for reversing the disruption of directional migration. However, recombinant reelin reduced the migration distance, which is in agreement with previous studies^40,41^, assuming that it acted on neurons. A previous study reported that human neural stem cells expressing reelin can migrate even if they are injected in the brain of *reeler* mice^40^. Taken together, our findings may suggest the importance of endogenous reelin expression for directional migration in human neurons. Further studies are warranted to confirm our findings.

While we identified a common phenotype in both congenital and isogenic RELN-del neurons, we also found some differences between them. For example, decreased dopamine production was observed only in congenital RELN-del neurons. Considering that the differentiation efficiency into TH+ neurons was similar (>85%) among all RELN-del iPSCs, the intracellular degradation of dopamine may be promoted in congenital RELN-del neurons. We have not identified the additional RELN-del subjects in more than 2000 SCZ patients. The small number of subjects carrying rare *RELN* variant is the limitation of our study. To elucidate a direct causal link between neuronal phenotypes and rare genetic variants such as *RELN*, the derivation of both isogenic and patient-specific iPSCs, such as those in this study, should prove to be a promising strategy.

The neurosphere outgrowth assay is an established assay for measuring neuronal migration ability^21^; however, most studies using this assay focus on migration distance. Our single-cell trajectory analysis demonstrated that healthy control iPSC-derived neurons demonstrate strictly controlled migration even in vitro, particularly with regard to direction. As we used highly homogenous neurons, there seems to be few signals from other cell types such as a glia in this system. Thus, our findings will be useful for addressing direct neuron dynamics at single-cell levels in humans. In addition to our study, some reports have demonstrated that iPSC-derived neurons show migration ability similar to that in vivo^42,43^. Although it should be noted that iPSC-derived neurons do not completely recapitulate the physiology of the human brain, they are undoubtedly full of novel findings about human brain dynamics.

Newborn neurons including dopaminergic neurons in midbrain migrate radially as well as neurons in cerebrum and cerebellum during brain development^44–47^. In general, migrating neurons are aligned along the radial glia, while we observed that iPSC-derived neurons could independently migrate from the radial glia. Our findings suggest that reelin widely plays a role in brain development, and may be useful for studying the mechanisms underlying neuronal migration independent of the radial glia in humans.

Clarifying the genetic, molecular, and cellular mechanisms underlying disease susceptibility is essential for the development of effective treatments. In this study, we demonstrated that a rare *RELN* variant causes an impaired reelin signal, resulting in a loss of directionality during individual human neuronal migration. We believe that our analysis, using both isogenic and congenital RELN-del iPSCs, made it possible to come to this conclusion. Moreover, our automatic detection system demonstrated the utility of RELN-del iPSCs for screening and evaluating human neurodevelopmental impairment. Our study, focusing on the directionality of individual cell migration, provides a foundation for the elucidation of reelin function in neural development and may contribute to novel therapeutic strategies for mitigating mental disorder susceptibility.

## Electronic supplementary material

Supplementary Figure

Supplementary Figure legends

Supplementary methods

Supplementary Table 1

Supplementary Table 2 and 6

Supplementary Table 3

Supplementary Table 4

Supplementary Table 5

## References

[1] Kushima I (2017). High-resolution copy number variation analysis of schizophrenia in Japan. Mol. Psychiatry.

[2] De Rubeis S (2014). Synaptic, transcriptional and chromatin genes disrupted in autism. Nature.

[3] Tissir F, Goffinet AM (2003). Reelin and brain development. Nat. Rev. Neurosci..

[4] Hong SE (2000). Autosomal recessive lissencephaly with cerebellar hypoplasia is associated with human RELN mutations. Nat. Genet..

[5] Ishii K, Kubo KI, Nakajima K (2016). Reelin and neuropsychiatric disorders. Front. Cell. Neurosci..

[6] Folsom TD, Fatemi SH (2013). The involvement of Reelin in neurodevelopmental disorders. Neuropharmacology.

[7] Impagnatiello F (1998). A decrease of reelin expression as a putative vulnerability factor in schizophrenia. Proc. Natl. Acad. Sci. USA.

[8] Lammert DB, Howell BW (2016). RELN mutations in autism spectrum disorder. Front. Cell. Neurosci..

[9] Laviola G, Ognibene E, Romano E, Adriani W, Keller F (2009). Gene-environment interaction during early development in the heterozygous reeler mouse: clues for modelling of major neurobehavioral syndromes. Neurosci. Biobehav. Rev..

[10] Kawauchi T (2015). Cellullar insights into cerebral cortical development: focusing on the locomotion mode of neuronal migration. Front. Cell. Neurosci..

[11] Evsyukova I, Plestant C, Anton ES (2013). Integrative mechanisms of oriented neuronal migration in the developing brain. Annu. Rev. Cell Dev. Biol..

[12] Valiente M, Marin O (2010). Neuronal migration mechanisms in development and disease. Curr. Opin. Neurobiol..

[13] Sharaf A, Rahhal B, Spittau B, Roussa E (2015). Localization of reelin signaling pathway components in murine midbrain and striatum. Cell Tissue Res..

[14] Howes OD, Kapur S (2009). The dopamine hypothesis of schizophrenia: version III—the final common pathway. Schizophr. Bull..

[15] Davis KL, Kahn RS, Ko G, Davidson M (1991). Dopamine in schizophrenia: a review and reconceptualization. Am. J. Psychiatry.

[16] Takahash K (2007). Induction of pluripotent stem cells from adult human fibroblasts by defined factors. Cell.

[17] Mayshar Y (2010). Identification and classification of chromosomal aberrations in human induced pluripotent stem cells. Cell Stem Cell.

[18] Martins-Taylor K (2011). Recurrent copy number variations in human induced pluripotent stem cells. Nat. Biotech..

[19] Amps K (2011). Screening ethnically diverse human embryonic stem cells identifies a chromosome 20 minimal amplicon conferring growth advantage. Nat. Biotechnol..

[20] Fujimori K (2016). Modeling neurological diseases with induced pluripotent cells reprogrammed from immortalized lymphoblastoid cell lines. Mol. Brain.

[21] Brennand K (2015). Phenotypic differences in hiPSC NPCs derived from patients with schizophrenia. Mol. Psychiatry.

[22] Lilja J (2017). SHANK proteins limit integrin activation by directly interacting with Rap1 and R-Ras. Nat. Cell Biol..

[23] Gorelik R, Gautreau A (2014). Quantitative and unbiased analysis of directional persistence in cell migration. Nat. Protoc..

[24] Sbalzarini IF, Koumoutsakos P (2005). Feature point tracking and trajectory analysis for video imaging in cell biology. J. Struct. Biol..

[25] Li HL, Gee P, Ishida K, Hotta A (2016). Efficient genomic correction methods in human iPS cells using CRISPR-Cas9 system. Methods.

[26] Stemmer M, Thumberger T, Del Sol Keyer M, Wittbrodt J, Mateo JL (2015). CCTop: an intuitive, flexible and reliable CRISPR/Cas9 target prediction tool. PLoS ONE.

[27] Bock HH, May P (2016). Canonical and non-canonical Reelin signaling. Front. Cell. Neurosci..

[28] Hook V (2014). Human iPSC neurons display activity-dependent neurotransmitter secretion: aberrant catecholamine levels in schizophrenia neurons. Stem Cell Rep..

[29] Robicsek O (2013). Abnormal neuronal differentiation and mitochondrial dysfunction in hair follicle-derived induced pluripotent stem cells of schizophrenia patients. Mol. Psychiatry.

[30] Mariani J (2012). Modeling human cortical development in vitro using induced pluripotent stem cells. Proc. Natl. Acad. Sci. USA.

[31] Mariani J (2015). FOXG1-dependent dysregulation of GABA/Glutamate neuron differentiation in autism spectrum disorders. Cell.

[32] Marchetto MC (2010). A model for neural development and treatment of Rett syndrome using human induced pluripotent stem cells. Cell.

[33] Imaizumi K (2015). Controlling the regional identity of hPSC-derived neurons to uncover neuronal subtype specificity of neurological disease phenotypes. Stem Cell Rep..

[34] Lee GH, D'Arcangelo G (2016). New insights into Reelin-mediated signaling pathways. Front. Cell. Neurosci..

[35] Fromer M (2014). De novo mutations in schizophrenia implicate synaptic networks. Nature.

[36] Zhou Z (2016). Identification of RELN variation p.Thr3192Ser in a Chinese family with schizophrenia. Sci. Rep..

[37] Costain G (2013). Pathogenic rare copy number variants in community-based schizophrenia suggest a potential role for clinical microarrays. Hum. Mol. Genet..

[38] Fatemi SH (2005). Reelin glycoprotein: structure, biology and roles in health and disease. Mol. Psychiatry.

[39] Imai H (2017). Dorsal forebrain-specific deficiency of Reelin-Dab1 signal causes behavioral abnormalities related to psychiatric disorders. Cereb. Cortex.

[40] Kim HM (2002). Reelin function in neural stem cell biology. Proc. Natl. Acad. Sci. USA.

[41] Dulabon L (2000). Reelin binds alpha3beta1 integrin and inhibits neuronal migration. Neuron.

[42] Bamba Y, Kanemura Y, Okano H, Yamasaki M (2017). Visualization of migration of human cortical neurons generated from induced pluripotent stem cells. J. Neurosci. Methods.

[43] Xiang Y (2017). Fusion of regionally specified hPSC-derived organoids models human brain development and interneuron migration. Cell. Stem. Cell..

[44] Kawano H, Ohyama K, Kawamura K, Nagatsu I (1995). Migration of dopaminergic neurons in the embryonic mesencephalon of mice. Brain. Res. Dev. Brain Res..

[45] Bodea GO (2014). Reelin and CXCL12 regulate distinct migratory behaviors during the development of the dopaminergic system. Development.

[46] Chedotal A (2010). Should I stay or should I go? Becoming a granule cell. Trends Neurosci..

[47] Lui JH, Hansen DV, Kriegstein AR (2011). Development and evolution of the human neocortex. Cell.

