# Supplementary material for: Single-cell trajectory analysis of human homogenous neurons carrying a rare RELN variant
Source: Transl Psychiatry. 2018 Jul 19;8:129. doi: 10.1038/s41398-018-0177-8 (PMC6052151; doi:10.1038/s41398-018-0177-8)
Supplement: Supplementary file 1 — Supplementary Figure [file 41398_2018_177_MOESM1_ESM.pdf]

a

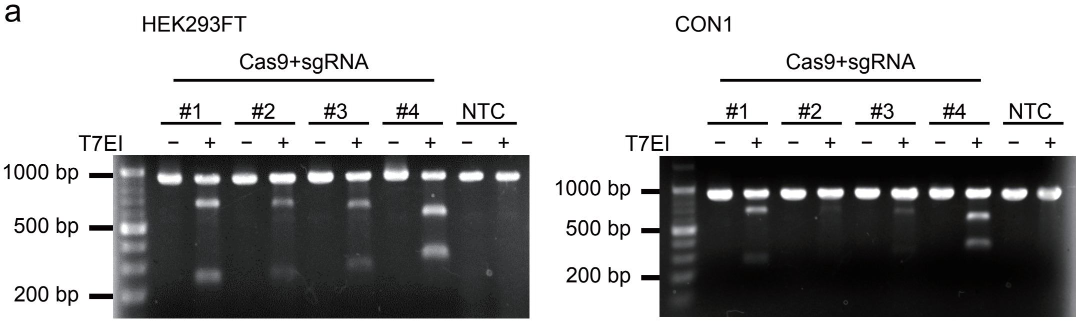

b

	Sequence	indel	genotype
source	ATCTGTATTCTTTCCA ACTAAAGGGTGGAAAAGGATCACCTACCCACTTCCTGAAAGCTTAG		
CON1	ATCTGTATTCTTTCCA ACTAAAGGGTGGAAAAGGATCACCTACCCACTTCCTGAAAGCTTAG<br>ATCTGTATTCTTTCCA - - - - AAGGGTGGAAAAGGATCACCTACCCACTTCCTGAAAGCTTAG	WT<br>−4bp	+/−
	ATCTGTA - - - - - - - - - - - - - - AGGGTGGAAAAGGATCACCTACCCACTTCCTGAAAGCTTAG<br>ATCTGT - - - - - - - - - - - GAAAAGGGTGGAAAAGGATCACCTACCCACTTCCTGAAAGCTTAG	−14bp<br>−11bp	−/−
CON2	ATCTGTATTCTTTCCA ACTAAAGGGTGGAAAAGGATCACCTACCCACTTCCTGAAAGCTTAG<br>ATCTGTATTCTTTCCA - - - - - - - - - - - - - - AAGGATCACCTACCCACTTCCTGAAAGCTTAG	WT<br>−14bp	+/−
	ATCTGTATTCTTTCCA - - - - - AGGGTGGAAAAGGATCACCTACCCACTTCCTGAAAGCTTAG<br>ATCTGTATTCTTTCCA - - - - - AGGGTGGAAAAGGATCACCTACCCACTTCCTGAAAGCTTAG	−5bp<br>−5bp	−/−

c

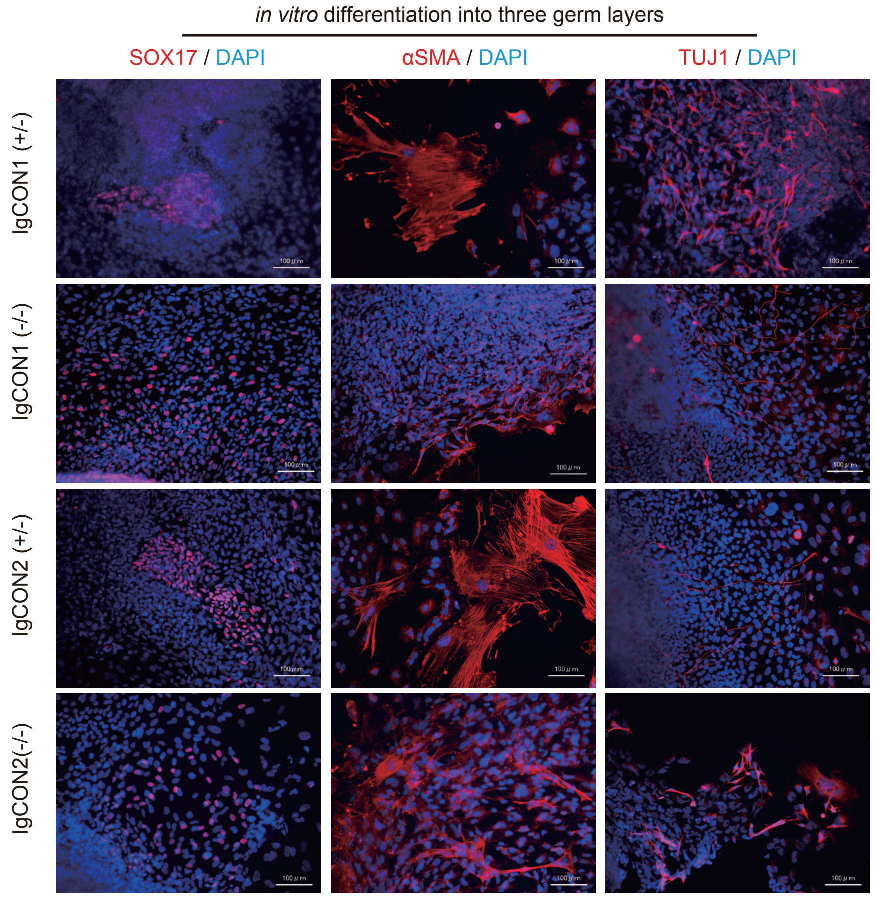

Supplementary Figure 1 Arioka et al

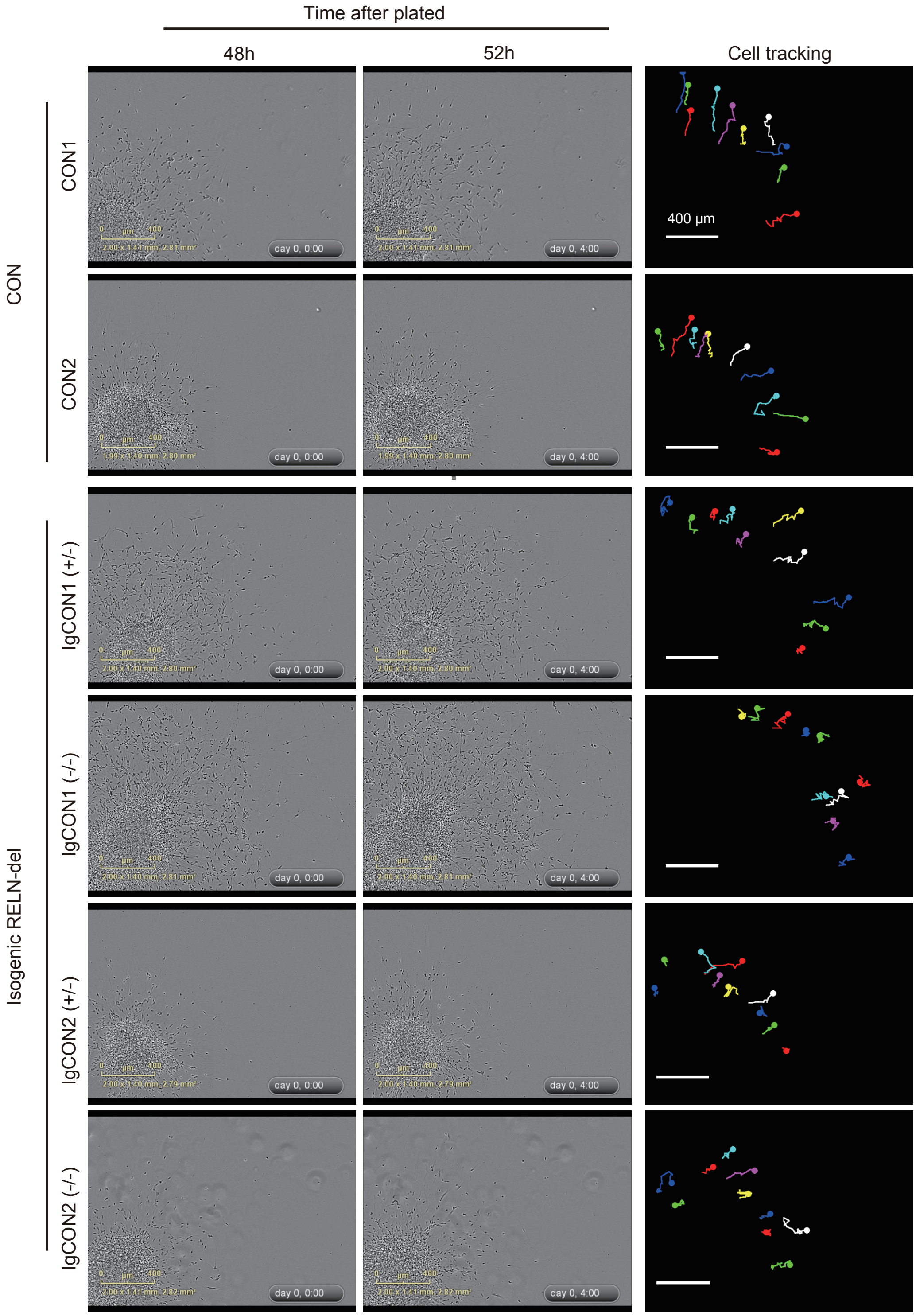

Supplementary Figure 2 Arioka et al

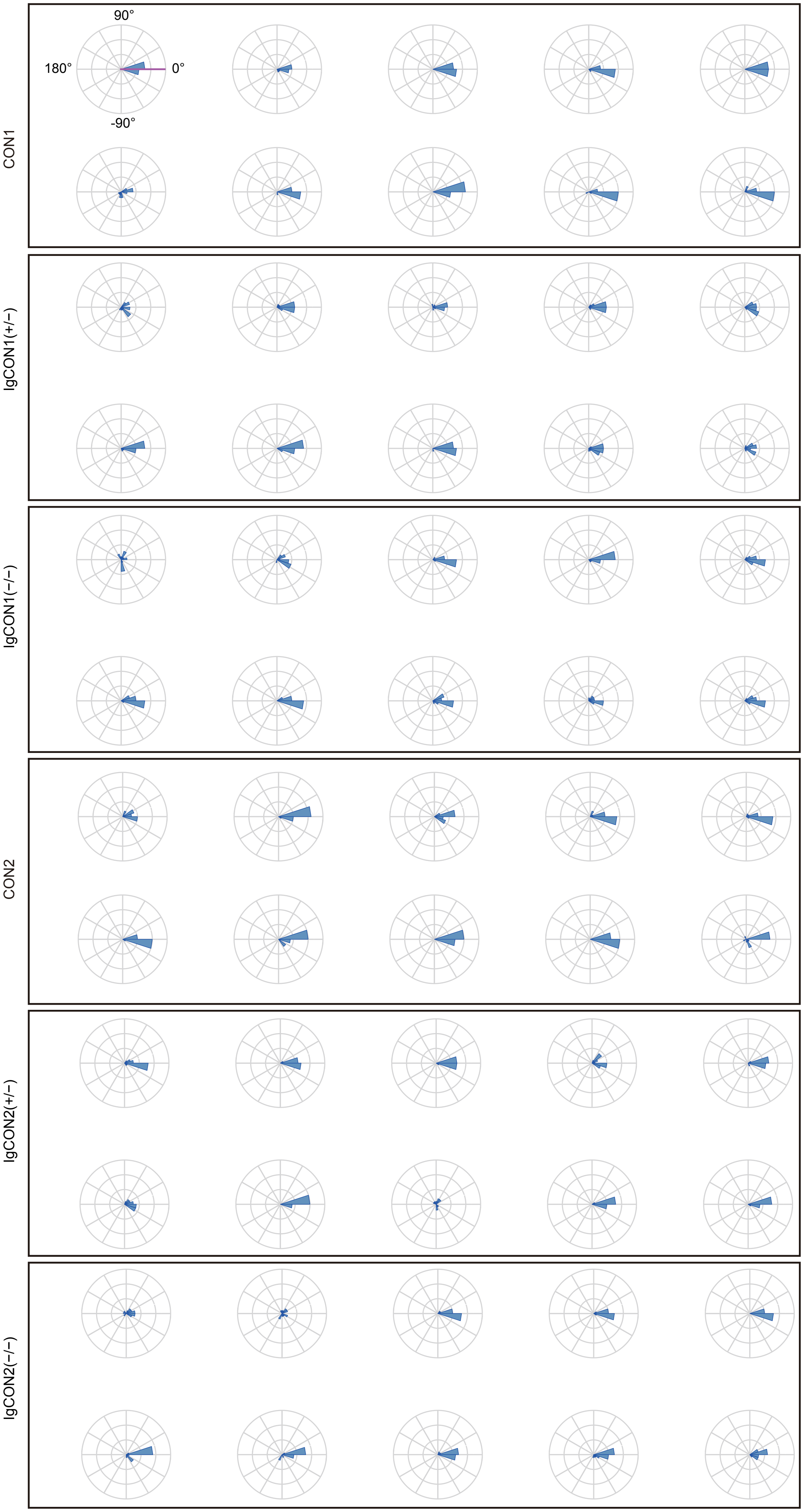

Supplementary
Figure 3
Arioka et al

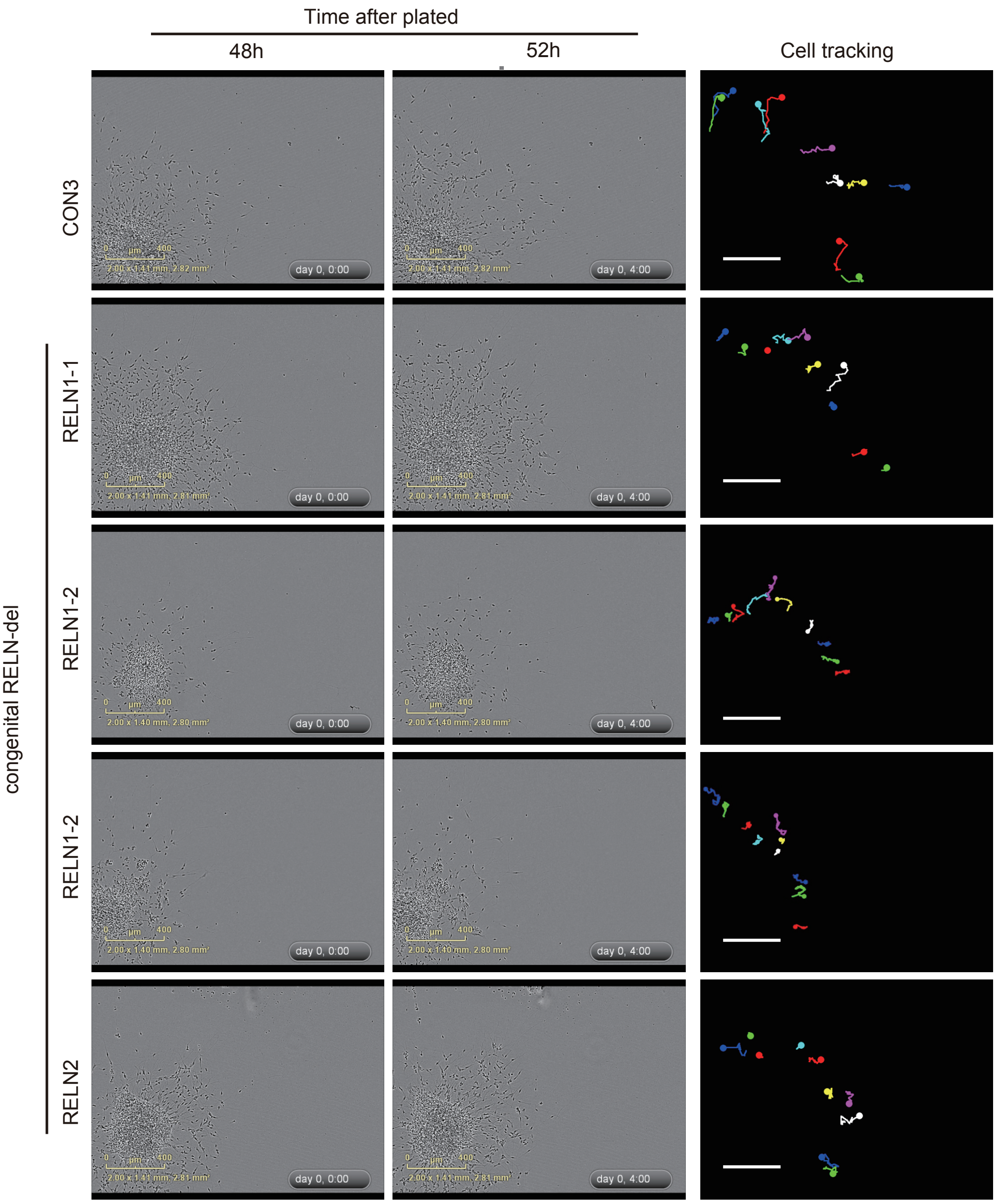

Supplementary Figure 4 Arioka et al

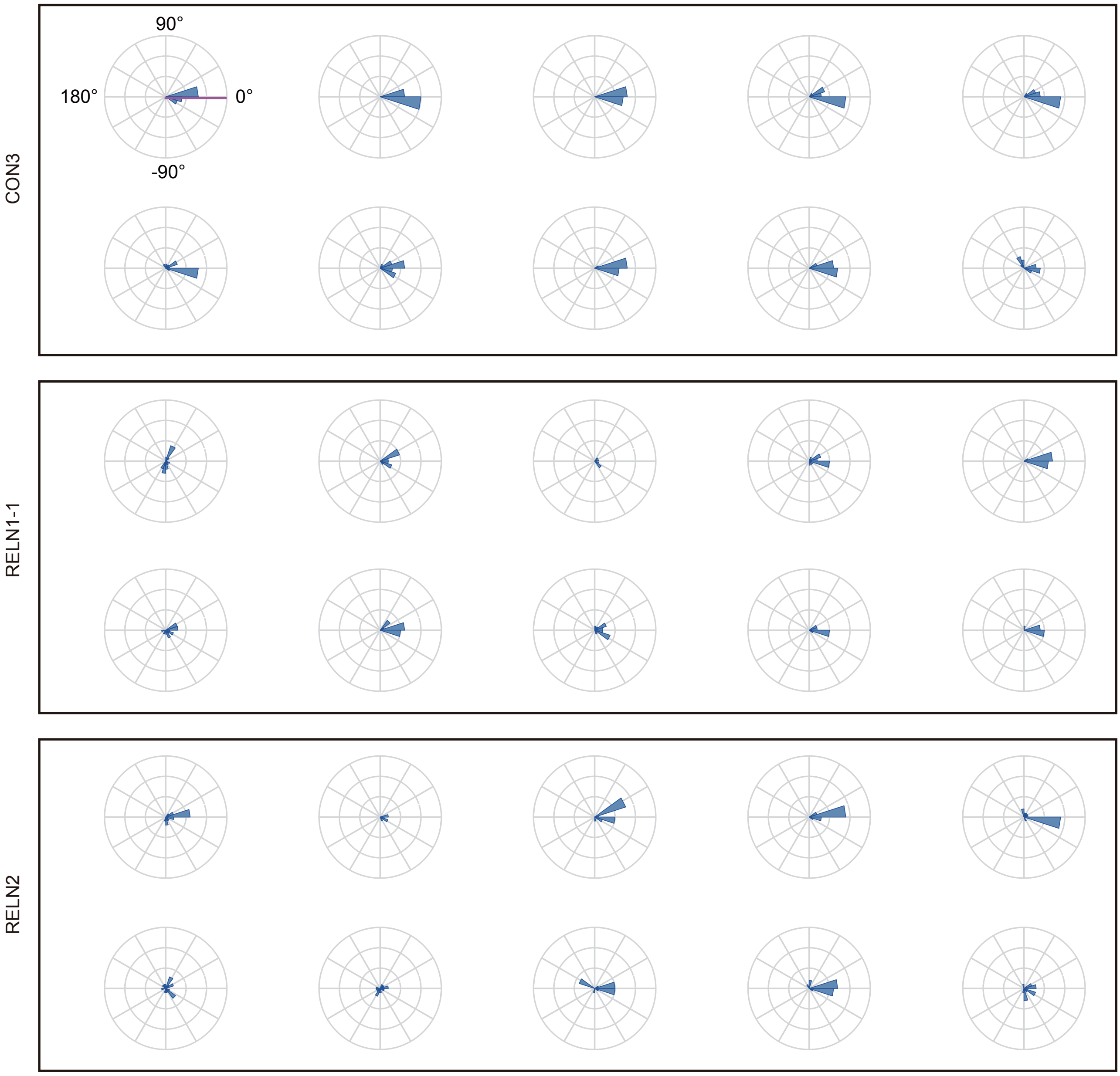

Supplementary Figure 5 Arioka et al

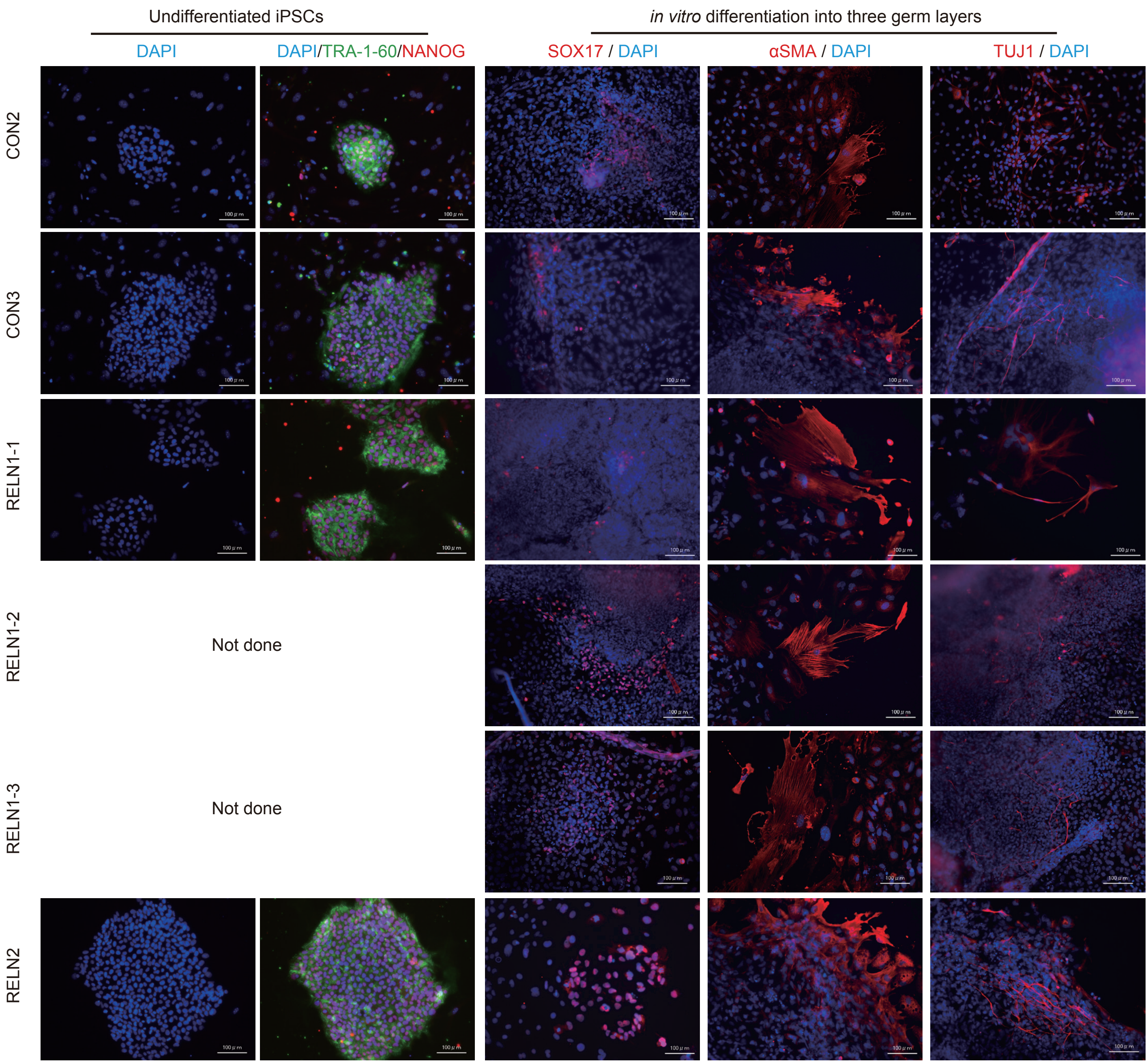

Supplementary Figure 6 Arioka et al
