# Supplementary material for: Single-cell trajectory analysis of human homogenous neurons carrying a rare RELN variant
Source: Transl Psychiatry. 2018 Jul 19;8:129. doi: 10.1038/s41398-018-0177-8 (PMC6052151; doi:10.1038/s41398-018-0177-8)
Supplement: Supplementary file 2 — Supplementary Figure legends [file 41398_2018_177_MOESM2_ESM.docx]

**Supplementary Figure legends**

**Supplementary Figure 1. Establishment of isogenic RELN-del lines.**

(a) Analysis of CRISPR-sgRNA activities by T7EI assay (HEK293FT and CON1). NTC: no transfection. (b) Indel patterns of RELN-del isogenic lines using CRISPR-sgRNA#4. CON1 and C ON2 were used as parental iPSCs. WT: wild-type sequence. Red letters represent stop codon. (c) Evaluation of the capacity for isogenic RELN-del lines to differentiate into all three germ layers. Bars represent 100 μm.

**Supplementary Figure 2. Real time imaging analysis of neuronal migration using isogenic RELN-del lines.**

*Left*: Phase contrast images at 48 h and 52 h after plating neurospheres on Matrigel-coated dishes with neuronal induction medium. *Right*: Cell tracking results from left panels. Bars represent 400 μm.

**Supplementary Figure 3. Distribution of cell movement cell angles for genotypes plotted on circular histograms.**

Results for CON1, CON2, IgCON1(+/−), IgCON1(−/−), IgCON2(+/−) and IgCON2(−/−).

**Supplementary Figure 4. Real time imaging analysis of neuronal migration using congenital RELN-del lines.**

*Left*: Phase contrast images at 48 h and 52 h after plating neurospheres on Matrigel-coated dishes with neuronal induction medium. *Right*: Cell tracking results from left panels. Bars represent 400 μm.

**Supplementary Figure 5. Distribution of cell movement cell angles for genotypes plotted on circular histograms.**

Results for CON3, RELN1-1 and RELN2.

**Supplementary Figure 6. Generated iPSCs express pluripotent markers and can differentiate into three germ layers.**

*Left*: Immunostaining for NANOG and TRA-1-60 in iPSC lines. *Right*: Evaluation of the capacity to differentiate into all three germ layers by SOX17 staining (endodermal marker), αSMA staining (mesodermal marker), and TUJ1 staining (ectodermal marker). Bars represent 100 μm.
