# Supplementary material for: Single-cell trajectory analysis of human homogenous neurons carrying a rare RELN variant
Source: Transl Psychiatry. 2018 Jul 19;8:129. doi: 10.1038/s41398-018-0177-8 (PMC6052151; doi:10.1038/s41398-018-0177-8)
Supplement: Supplementary file 3 — Supplementary methods [file 41398_2018_177_MOESM3_ESM.docx]

**Supplementary Methods**

**iPSC generation**

iPSCs were generated from the peripheral blood mononuclear cells of subjects CON1, CON2, RELN1, and RELN2 using episomal vectors, as previously described^1^. The use of human samples and the genomic analysis were approved by the Ethics Committee of Nagoya University (Approval Number: 2012-0184) and Keio University School of Medicine Ethics Committee (Approval Number: 20-16-18). Generated iPSCs were cultured on mitomycin-C-treated mouse embryonic fibroblast (MEF) feeder cells in iPSC medium consisting of Dulbecco’s Modified Eagles Medium (DMEM/F12) supplemented with 20% Knockout Serum Replacement (KSR, GIBCO, USA), 2 mM L-glutamine, 0.1 mM nonessential amino acids, 0.1 mM 2-mercaptoethanol, 100 units/mL penicillin, and 100 μg/mL streptomycin, and 4 ng/ml basic fibroblast growth factor (bFGF). For MEF feeder cell-free cultures, iPSCs were maintained on Matrigel (BD)-coated dishes with MEF-conditioned medium (the iPSC medium described was pre-exposed to MEF feeder cells overnight). All generated iPSCs showed the capacity to differentiate into three germ layers *in vitro* (Supplementary Fig. 6)

**CGH array and TaqMan copy number assays**

Extraction of genomic DNA from peripheral blood and iPSCs were outsourced to LSI Medience Corporation (Japan). Array Comparative genomic hybridization (aCGH) and TaqMan copy number assays were performed as referred to our previous study^5^ with some modification. We used Agilent SurePrint G3 human CGH 400k (Agilent, Santa Clara, CA) for aCGH. Copy number variation (CNV) calls were made with Nexus Copy Number software v8.0 (BioDiscovery, El Segundo, CA) using the Fast Adaptive States Segmentation Technique 2 algorithm. Three contiguous probes were required for CNV calls. The log2 ratio thresholds for CNV calls were −0.6 (loss) and 0.5 (gain). The following CNVs were excluded from the CNV list: 1) CNVs with > 50% overlap with CNVs reported in Database of Genomic Variants (DGV) database^2^ (i.e., CNVs in healthy controls), and 2) CNVs on the Y chromosome. All genomic locations are given in NCBI build36.

**Embryoid body (EB) formation and *in vitro* differentiation**

EB formation was performed as our previous study^3^. Briefly, iPSCs were dissociated by TrypLE select for 5 min, and then harvested in DMEM/F12 containing 5% KSR, 2 mM L-glutamine, 0.1 mM nonessential amino acids, 0.1 mM 2-mercaptoethanol, 10 μM Y27632, 100 units/mL penicillin and 100 μg/mL streptomycin. After seven days of floating culture, the EBs were transferred onto gelatin-coated plates and cultured in DMEM containing 10% fetal bovine serum (FBS) for another seven days to induce spontaneous differentiation.

**DNA constructs for clustered regularly interspaced short palindromic repeat (CRISPR) / CRISPR-associated 9 (Cas9) systems**

We generated each expression vector as in a previous study^4^. A Cas9 expression vector (pHL-EF1α-SphcCas9-iP) with a puromycin-resistant cassette was obtained from Addgene (ID: 60599). To construct sgRNA expression vectors, two oligos containing the sgRNA target site and a universal reverse primer (Supplementary Table 2) were amplified by PCR and cloned into the BamHI-EcoRI site of the pHL-H1-ccdB-mEF1α-RiH vector (Addgene ID: 60601) with a RFP cassette. All procedures were conducted in accordance with the guidelines for recombinant DNA research established by Nagoya University.

**Transfection of CRISPR into HEK293FT and human iPSCs**

HEK293FT cells were cultured in DMEM supplemented with 10% FBS, 100 units/mL penicillin, and 100 μg/mL streptomycin. The cells were co-transfected with Cas9 and sgRNA using Lipofectamine 3000 (Invitrogen, USA), according to the manufacturers’ instructions. Forty-eight hours after transfection, 2 μg/ml puromycin was added for selection.

For transfection of CRISPR into human iPSCs, feeder-free cultured CON1 and CON2-derived iPSCs were pretreated with Y-27632 at 10 μM for at least 1 h and dissociated into single cells by treatment with TrypLe Select for 5 min at 37°C. Suspension containing 1×10^6^ cells were then transfected with Cas9 and sgRNA using FuGENE HD (Promega, USA). Cells were plated onto one Matrigel-coated well of a six-well plate under feeder-free conditions in the presence of 10 μM Y-27632 for 24 h. Forty-eight hours after transfection, 2 μg/ml puromycin was added for selection. Twenty-four hours later, the cells were cultured in MEF-conditioned medium until they formed large colonies.

**T7 endonuclease I (T7EI) assay**

Puromycin-selected cells were used for T7EI assay to examine sgRNA activity. The target region of sgRNAs was amplified from genomic DNA by a high-fidelity PCR reaction using the following primers; Fw: ggcaatcatggtgactttctctg, Rv: cagagcaaggtttaatcacctctg. The PCR products were denatured (95°C for 2 min) and re-annealed (85°C–25°C at −0.1°C/s), followed by T7E1 (NEB) digestion for 30 min at 37°C. The digested products were analyzed on 1.5% agarose gels.

**Immunocytochemistry and immunoblotting**

For immunocytochemistry, cells were fixed in 4% paraformaldehyde (PFA) for 15 min, permeabilized and blocked in PBS containing 0.3% TritonX-100 and 1% BSA for 60 min, and then incubated with the indicated primary antibodies overnight at 4°C. After washing with PBS, immunolabelled cells were incubated with appropriate fluorophore-tagged secondary antibodies for 1 h at room temperature. The primary antibodies used in this study were as follows: anti-TRA-1-60 (abcam, UK); anti-NANOG (abcam); anti-SOX17 (R&D systems, USA); anti-αSMA (R&D systems); anti-TUJ1 (Sigma-Aldrich, USA); anti-TH (Millipore, USA); anti-FOX2A (R&D systems); and anti-Reelin (MBL, Japan). Images were captured using a BZ-9000 fluorescence microscope (KEYENCE, Japan) or LSM780 confocal microscope (Zeiss, Germany). For immunoblotting, DA neurons (Day 28) were used. Primary antibodies were as follows: anti -TH (Millipore); anti-phosphorylated DAB1 tyrosine (Y232) (CST, USA); anti-total DAB1 (CST, USA) and anti-β-Actin-Peroxidase (Sigma-Aldrich). Used secondary antibodies were anti-mouse or anti-rabbit horseradish peroxidase-conjugated antibodies (GE Healthcare, UK). Quantification of immunoblots was done with image J.

**Expression DNA microarray and quantitative polymerase chain reaction (qPCR)**

Total RNA was extracted using the RNeasy Plus Mini Kit (QIAGEN, Germany). DNA microarray analysis was performed using the SurePrint G3 Hmm GE 8x60K V2 Microarray Kit (Agilent Technology, USA), in accordance with the manufacturer’s instructions. Sample number for microarray was *n* =3. All data analyses were performed using the GeneSpring GX Software Program (version 13; Agilent Technology). Absolute expression values were normalized using quantile normalization. The statistical criteria for differential expression were as follows; moderated *t*-test, Benjamini-Hochberg FDR for multiple testing correction, corrected *p*-value < 0.05, and fold changes > 2. GO analysis (using GOTERM_BP_FAT) was performed with DAVID’s functional annotation tool (https://david.ncifcrf.gov/). Reverse transcription (RT) was performed using the High-Capacity cDNA Transcription Kit (Applied Biosystems). Gene expression analysis by qPCR was conducted using the 7900HT Fast Real-Time PCR System (Applied Biosystems, USA) and the KAPA SYBR Fast qPCR Kit (KAPA BIOSYSTEMS, USA). Primers used for RT-qPCR are listed in Supplementary Table 6.

**Recombinant reelin protein**

Full-length mouse reelin cDNA^5^ tagged with PA was cloned into phCMV3. The preparation and purification of reelin recombinant was performed as previously reported^6, 7^.

**Measurement of dopamine concentration in medium**

To evaluate the capacity of differentiated dopaminergic neurons to generate and release dopamine, we measured the dopamine concentration in media collected seven days after induction (Day 28) using an ELISA kit (DLD EA608/96, Germany). At least three independent experiments on separate cultures were performed.

1. Okita K, Matsumura Y, Sato Y, Okada A, Morizane A, Okamoto S *et al.* A more efficient method to generate integration-free human iPS cells. *Nature methods* 2011; **8**(5)**:** 409-412.

2. MacDonald JR, Ziman R, Yuen RK, Feuk L, Scherer SW. The Database of Genomic Variants: a curated collection of structural variation in the human genome. *Nucleic acids research* 2014; **42**(Database issue)**:** D986-992.

3. Arioka Y, Ito H, Hirata A, Semi K, Yamada Y, Seishima M. Behavior of leucine-rich repeat-containing G-protein coupled receptor 5-expressing cells in the reprogramming process. *Stem cell research* 2017; **20:** 1-9.

4. Li HL, Fujimoto N, Sasakawa N, Shirai S, Ohkame T, Sakuma T *et al.* Precise correction of the dystrophin gene in duchenne muscular dystrophy patient induced pluripotent stem cells by TALEN and CRISPR-Cas9. *Stem cell reports* 2015; **4**(1)**:** 143-154.

5. D'Arcangelo G, Nakajima K, Miyata T, Ogawa M, Mikoshiba K, Curran T. Reelin is a secreted glycoprotein recognized by the CR-50 monoclonal antibody. *The Journal of neuroscience : the official journal of the Society for Neuroscience* 1997; **17**(1)**:** 23-31.

6. Fujii Y, Kaneko M, Neyazaki M, Nogi T, Kato Y, Takagi J. PA tag: a versatile protein tagging system using a super high affinity antibody against a dodecapeptide derived from human podoplanin. *Protein expression and purification* 2014; **95:** 240-247.

7. Suzuki K, Tsunoda H, Omiya R, Matoba K, Baba T, Suzuki S *et al.* Structure of the Plexin Ectodomain Bound by Semaphorin-Mimicking Antibodies. *PloS one* 2016; **11**(6)**:** e0156719.
