# Supplementary material for: Single-cell trajectory analysis of human homogenous neurons carrying a rare RELN variant
Source: Transl Psychiatry. 2018 Jul 19;8:129. doi: 10.1038/s41398-018-0177-8 (PMC6052151; doi:10.1038/s41398-018-0177-8)
Supplement: Supplementary file 5 — Supplementary Table 2 and 6 [file 41398_2018_177_MOESM5_ESM.docx]

**Supplementary Table 2. Predicted off-target sites (Homo sapiens GRCh37/hg19)**

| Coodinates | strand | MM | Target sequence | PAM | position | Gene name |
| --- | --- | --- | --- | --- | --- | --- |
| chr7:103143496-103143518 | + | 0 | ATCCTTTT[CCACCCTTTAGT] | TGG | Exonic | RELN |
| chr14:82481472-82481494 | − | 3 | AT**AA**TTTT[C**T**ACCCTTTAGT] | GGG | Intronic | RP11-666E17.1 |
| chr2:133863979-133864001 | + | 3 | ATCCT**AC**T[**A**CACCCTTTAGT] | AGG | Intronic | AC011755.1 |
| chr5:112017584-112017606 | − | 3 | ATCCTTT**C**[**A**CACC**T**TTTAGT] | GGG | Intergenic | APC |
| chr9:19888144-19888166 | + | 3 | ATCCTTT**C**[C**A**ACC**T**TTTAGT] | GGG | Intergenic | RP11-378I6.1 |
| chr7:47448004-47448026 | − | 3 | ATCCT**G**T**C**[CCACCCT**G**TAGT] | GGG | Intronic | TNS3 |
| chr1:108075601-108075623 | − | 3 | ATCC**C**T**C**T[CCACCCTT**A**AGT] | GGG | Intergenic | RP11-270C12.3 |
| chr17:29011023-29011045 | − | 3 | ATCC**C**TTT[CC**T**CCCT**A**TAGT] | TGG | Intergenic | AC005562.1 |
| chr9:25926215-25926237 | − | 3 | ATC**T**TTTT[CCACC**T**TTTA**A**T] | TGG | Intergenic | RP11-477G9.1 |
| chr9:25926215-25926237 | − | 3 | ATC**T**TTTT[CCA**G**CCTTTAG**A**] | GGG | Intergenic | AC010980.2 |

**Supplementary Table 6. Primer sequence**

| Targets | Primers | Sequence (5’→3’) |
| --- | --- | --- |
| Construction of sgRNAs | sgRNA#1-Fw | GAGACCACTTGGATCCGTACTGACTTCGGTGTGAGTGTTTTAGAGCTAGAAATAGCA |
|  | sgRNA#2-Fw | GAGACCACTTGGATCCGGGTGTGAGTTGGAATTATCGTTTTAGAGCTAGAAATAGCA |
|  | sgRNA#3-Fw | GAGACCACTTGGATCCGCAGAGCATTTTGGGTCAGCGTTTTAGAGCTAGAAATAGCA |
|  | sgRNA#4-Fw | GAGACCACTTGGATCCGTCCTTTTCCACCCTTTAGTGTTTTAGAGCTAGAAATAGCA |
|  | sgRNA#universal-Rv | GCCCGGGTTTGAATTCAAAAAAAGCACCGACTCGGTGCCACTTTTTCAAGTTGATAACGGACTAGCCTTATTTTAACTTGCTATTTCTAGCTCTAA |
| *RELN* | RELN-Fw | AAGGACAAGACTCACAATGCTC |
|  | RELN-Rv | CCAGCATTACGGAATGAAGGTC |
| *RPS18* | RPS18-Fv | GCGGCGGAAAATAGCCTTTG |
|  | RPS18-Rv | GATCACACGTTCCACCTCATC |
